# Mapping Neurodegenerative Changes in Clinically Uncertain Parkinsonian Syndrome Patients Using Fast MR Spin TomogrAphy in Time‐Domain (MR‐STAT) Relaxometry at 3T

**DOI:** 10.1002/jmri.70349

**Published:** 2026-04-25

**Authors:** Martin B. Schilder, Elon D. Wallert, Stefano Mandija, Oscar van der Heide, Hongyan Liu, Miha Fuderer, Edwin Versteeg, Jan Booij, Rob M. A. de Bie, Martijn Beudel, Henk W. Berendse, Tom van Mierlo, Jeroen Blankevoort, Cornelis A. T. van den Berg, Elsmarieke van de Giessen, Alessandro Sbrizzi

**Affiliations:** ^1^ Computational Imaging Group for MRI Therapy & Diagnostics, Department of Radiotherapy University Medical Center Utrecht Utrecht the Netherlands; ^2^ Department of Radiology and Nuclear Medicine, Amsterdam UMC University of Amsterdam Amsterdam the Netherlands; ^3^ Department of Neurology, Amsterdam Neuroscience, Amsterdam UMC University of Amsterdam Amsterdam the Netherlands; ^4^ Department of Neurology, Amsterdam UMC Vrije Universiteit Amsterdam the Netherlands; ^5^ Department of Neurology Spaarne Gasthuis Haarlem the Netherlands; ^6^ Department of Neurology Flevoziekenhuis Almere the Netherlands; ^7^ Department of Radiology and Nuclear Medicine, Amsterdam UMC Vrije Universiteit Amsterdam the Netherlands; ^8^ Amsterdam Neuroscience Brain Imaging Amsterdam the Netherlands

**Keywords:** CUPS, MR‐STAT, parkinsonism, quantitative MRI, relaxometry

## Abstract

**Background:**

MR Spin TomogrAphy in Time‐domain (MR‐STAT) enables accelerated multiparametric relaxometry (T_1_/T_2_). Previous relaxometry studies predominantly compared Parkinson's disease patients with healthy controls (HC). The potential of relaxometry to distinguish neurodegenerative from non‐neurodegenerative parkinsonism in clinically uncertain parkinsonian syndrome (CUPS) patients is unclear.

**Purpose:**

To investigate T_1_‐/T_2_‐differences between neurodegenerative and non‐neurodegenerative parkinsonism in CUPS patients.

**Study Type:**

Prospective cross‐sectional study.

**Population:**

52 patients with neurodegenerative and 57 patients with non‐neurodegenerative parkinsonism, diagnosed via dopamine transporter single photon emission computed tomography (DAT SPECT) and neurologist review, and 10 HC.

**Fieldstrength/Sequence:**

MP‐RAGE (magnetization‐prepared rapid acquisition with gradient‐echoes) and MR‐STAT, a 2D Cartesian‐encoded gradient‐spoiled gradient‐echo sequence with time‐varying flip‐angle preceded by a non‐selective inversion pulse, at 3T.

**Assessment:**

Repeatability of T_1_‐/T_2_‐values was evaluated for cortical gray matter/cerebral white matter/thalamus/putamen/caudate nucleus/globus pallidus (GP) in HC. T_1_‐_/_T_2_‐values of the parkinsonism groups were compared in the same regions per most/less affected hemisphere (MAH/LAH), determined by the putaminal uptake ratio on DAT SPECT.

**Statistical Tests:**

Regional coefficients of variation (CoV) were computed to assess the repeatability of T_1_‐/T_2_‐values in HC. T‐tests (*α* = 0.05) were used to compare T_1_−/T_2_‐values between parkinsonism groups, and Cohen's D values were computed with bootstrapping to measure effect sizes with 95% confidence intervals (95% CI).

**Results:**

CoVs ranged from 0.5% to 1.7% (T_1_) to 1.5% to 2.7% (T_2_). In the MAH, significant T_1_‐differences were found in the thalamus (Cohen's D = 0.635, 95% CI = [0.251, 1.016]); GP (Cohen's D = 0.508, 95% CI = [0.129, 0.887]); internal GP (Cohen's D = 0.603, 95% CI = [0.220, 0.983]); external GP (Cohen's D = 0.411, 95% CI = [0.033, 0.787]); and centromedial putamen (Cohen's D = 0.447, 95% CI = [0.069, 0.824]). In the LAH, significant T_1_‐differences were found in the thalamus (Cohen's D = 0.476, 95% CI = [0.097, 0.853]); GP (Cohen's D = 0.415, 95% CI = [0.037, 0.791]); anteromedial putamen (Cohen's D = 0.388, 95% CI = [0.011, 0.764]); and external GP (Cohen's D = 0.416, 95% CI = [0.038, 0.792]). T_2_‐differences were non‐significant.

**Data Conclusion:**

MR‐STAT showed high repeatability and showed potential to differentiate neurodegenerative from non‐neurodegenerative parkinsonism in CUPS patients.

**Evidence Level:**

1.

**Technical Efficacy:**

1.

## Plain Language Summary

This study tested a fast MRI method called MR‐STAT to measure brain tissue changes in people with an uncertain diagnosis of Parkinson's disease. The diagnosis is important, because treatment strategies depend on this distinction. When comparing patients with neurodegenerative causes versus non‐neurodegenerative causes, we found shorter clear differences between the two groups in specific deep brain regions. These findings suggest that MR‐STAT could in the future support the diagnosis.

## 1 Introduction

Parkinsonism is a clinical syndrome defined by bradykinesia in combination with rigidity, rest tremor, or both [1]. The most common cause is Parkinson's disease (PD), a neurodegenerative disorder characterized by progressive loss of dopaminergic neurons of the nigrostriatal pathway [1, 2]. However, parkinsonian symptoms can also result from non‐neurodegenerative conditions such as drug‐induced parkinsonism or vascular parkinsonism [1, 3]. Distinguishing neurodegenerative from non‐neurodegenerative parkinsonism can be challenging, especially in the early stages of the disease or in patients with atypical clinical features, as symptoms may overlap.

In cases of clinically uncertain parkinsonian syndromes (CUPS), clinical MRI is frequently performed to exclude structural brain abnormalities. However, MRI sequences in common practice are not designed to visualize the dopaminergic neurodegenerative changes characteristic of PD and therefore do not directly aid the diagnosis [4]. When clinical uncertainty persists after MRI, presynaptic dopaminergic imaging like dopamine transporter (DAT) single photon emission computed tomography (SPECT) or positron emission tomography (PET) can be used to assess nigrostriatal integrity to support in the clinical diagnosis [5]. These nuclear imaging techniques, however, are not always available and they are expensive [6, 7, 8]. Since most patients with CUPS undergo MRI as part of their diagnostic work‐up, the development of MRI‐derived markers may potentially improve the diagnostic process with only limited additional costs.

Recent advances in quantitative MRI, particularly relaxometry‐based techniques, have enabled widely available and non‐invasive assessments and may provide additional diagnostic value in patients with parkinsonism [8, 9, 10, 11, 12]. For instance, it has been demonstrated that T_1_‐values in various cortical and subcortical gray matter (GM) regions are shorter in patients with PD than controls [8]. Additionally, spatially dependent differences in estimated T_1_‐values in the putamen have been observed [9], and one study has reported longer T_1_‐values in patients with PD [12]. Furthermore, using higher‐resolution multiparametric quantitative protocols, previous studies have reported shorted T_1_‐values in the substantia nigra of PD patients [10, 11].

However, these studies have included only patients with a confirmed diagnosis of PD and have compared them to healthy age‐matched controls. Patients with an uncertain diagnosis were not included. Therefore, the diagnostic value of these novel imaging techniques in patients with CUPS, who may benefit most, is unclear.

Thus the aim of this study was to assess regional brain relaxometry differences in patients with CUPS, comparing patients with a final diagnosis of dopaminergic neurodegenerative versus non‐neurodegenerative parkinsonism, using MR Spin TomogrAphy in Time‐domain (MR‐STAT) relaxometry [13], a fast transient‐state multiparametric MRI technique that quantitatively maps the T_1_‐ and T_2_‐properties of brain tissue, either for synthetic contrasts generation [14] or quantitative data analysis [15].

## 2 Methods

This study was approved by the institutional review board of Amsterdam UMC and registered in the ‘Overview of Medical Research in the Netherlands’ (formerly the Dutch Trial Register; registration number NL79240.018.22, https://onderzoekmetmensen.nl/en/trial/54005). Patients and healthy controls (HC) were included in accordance with the principles outlined in the Declaration of Helsinki. All patients and healthy controls gave written informed consent prior to scanning.

### 2.1 MRI Acquisition and Data Processing

MRI acquisitions were performed on a 3T Ingenia scanner (Philips Healthcare, Best, The Netherlands) using a clinical 32‐channel receiver head coil (Philips Healthcare, Best, The Netherlands). Both a multi‐2D MR‐STAT scan and a 3D Magnetization Prepared Rapid Gradient Echo (MP‐RAGE) scan (for reference) were acquired. The acquisition parameters are listed in Table 1. The MR‐STAT sequence consisted of 30 Cartesian‐encoded 2D slices. Each slice was sequentially acquired with a gradient spoiled gradient echo scheme with a slowly varying flip angle preceded by a non‐selective inversion pulse, with an acquired and reconstructed resolution of 1 × 1 × 3 mm^3^ and a 1.5 mm interslice gap. Scan time for MR‐STAT was less than 5 min. The sequence was optimized using methods from Fuderer et al. [16] and the resulting flip angle train is provided in Supporting Information S1. The reconstruction was performed according to methods presented in van der Heide et al. [17] and included a B_1_‐field correction using the dual refocusing echo acquisition mode (DREAM) sequence [18]. The offline reconstruction time was approximately 2 min per slice on an NVIDIA RTX A5000 GPU with 24GB VRAM.

**TABLE 1:** Acquisition parameters of the multiparametric quantitative mapping sequence and the anatomical reference sequence.

	2D MR‐STAT	3D MP‐RAGE
FOV (AP × LR × CC) [mm]	224 × 224 × 133.5	256 × 180 × 256
Matrix size (AP × LR × CC)	224 × 224 × 30	256 × 180 × 256
Voxel size [mm^3^]	1 × 1 × 3	1 × 1 × 1
Interslice gap [mm]	1.5	0
TR [ms]	8.5	6.9
TE [ms]	4.1	3.1
Flip angle [deg]	Variable, see Supporting Information: 1.	9
Scan time [min:sec]	4:46	5:49

Abbreviations: AP = anterior–posterior; CC = cranio‐caudal; FOV = field‐of‐view; LR = left–right; MP‐RAGE = magnetization prepared rapid gradient echo; MR‐STAT = magnetic resonance spin tomography in time‐domain; TE = echo time; TR = repetition time.

Automatic brain tissue segmentations were performed using the reference MP‐RAGE images as input for AssemblyNet [19]. The following masks per hemisphere were selected: cortical gray matter (GM), cerebral white matter (WM), thalamus, putamen, caudate nucleus, and globus pallidus. Due to the limited spatial resolution and interslice gap in the craniocaudal direction, the substantia nigra, which is a potentially relevant structure to image in studies around parkinsonism [10, 11], was not included in the analyses.

**FIGURE 1:**
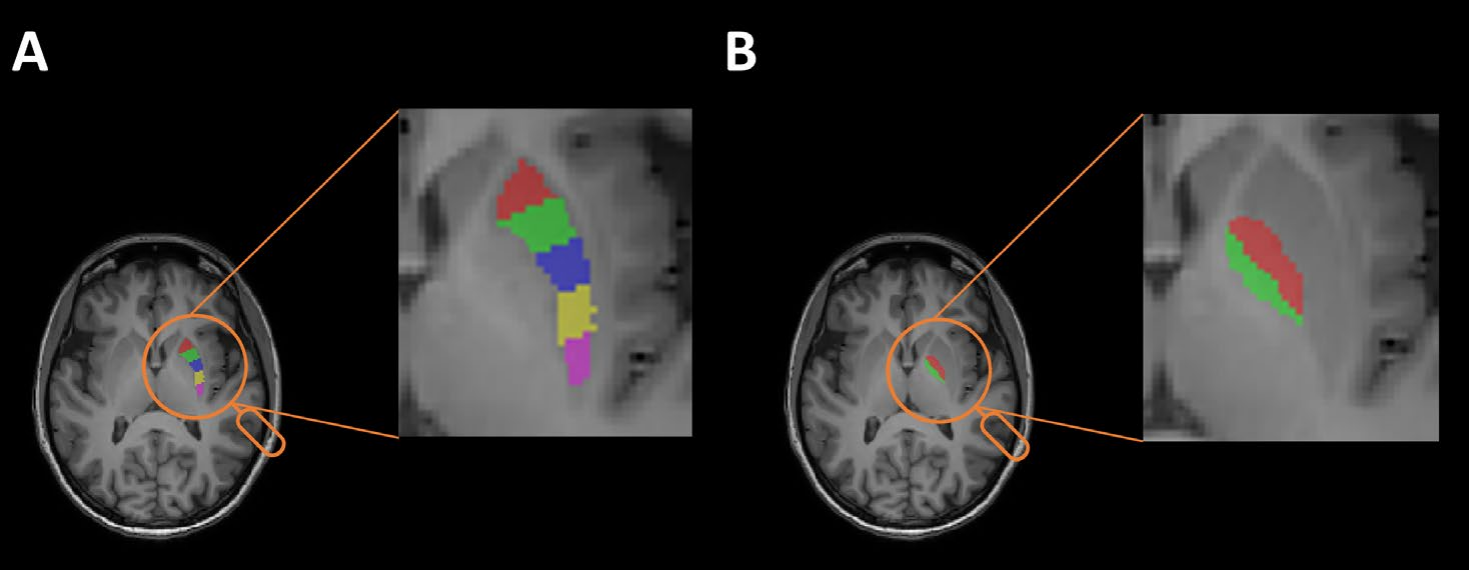
Example of segmentations. (A) Anterior (red), anteromedial (green), centromedial (blue), posteromedial (yellow), posterior (pink) putamen partitions; (B) Internal (green) and external (red) globus pallidus.

The masks of the putamen and the globus pallidus were automatically further partitioned using principal component analysis (PCA) methods described in Drori et al. [9], the putamen was split into 5 partitions. In our study, 5 partitions were used instead of 7 due to limited spatial resolution. Similar to the method used to partition the putamen, the globus pallidus was partitioned into an internal and external compartment using PCA to approximate the internal globus pallidus and external globus pallidus, as a previous study has suggested differential involvement of these subdivisions in parkinsonism [20]. Figure 1 shows an example of segmentation of the putamen partitions and the segmentation of the internal and external globus pallidus.

The rigid co‐registration was performed in MATLAB (Image Processing Toolbox) using the function imregtform to compute the transformation matrix from the MP‐RAGE frame of reference to the MR‐STAT frame of reference. The resulting transformation matrix was then applied to the masks using imwarp with nearest‐neighbor interpolation to preserve label integrity (MATLAB 2023A, The MathWorks Inc., Nattick, MA, USA).

### 2.2 In Vivo Repeatability

To assess the test–retest consistency of T_1_‐ and T_2_‐measurements, an in vivo repeatability study was conducted using the 2D MR‐STAT protocol. Ten HCs (4 males; 6 females; mean age: 29.6 years; range: 24–36 years) were enrolled. The MR‐STAT sequence was planned based on an MP‐RAGE scan and covered the entire brain. Following the first scan, participants were removed from the scanner and asked to stand before being repositioned for the second scan.

For each of the HCs, the mean T_1_‐ and T_2_‐values of the GM, WM, thalamus, putamen, caudate nucleus, and globus pallidus of both scanning sessions were computed using segmentation and registration methods described above.

### 2.3 Imaging Biomarkers in Neurodegenerative Versus Non‐Neurodegenerative Patients

The primary aim of this study was to assess differences in relaxation times between patients with dopaminergic neurodegenerative and non‐neurodegenerative parkinsonism in a cohort of patients with CUPS. Therefore, patients with CUPS in whom the treating neurologist requested a DAT SPECT scan to establish a diagnosis after January 1, 2019 were retrospectively identified. Patients who visited the neurology outpatient clinics of Amsterdam UMC, Spaarne Gasthuis, or Flevohospital were invited to participate. Individuals with contraindications to MRI or those unable to provide informed consent were excluded.

After obtaining written informed consent, a single MR‐STAT acquisition was performed using the same MRI protocol as described above. The patient's electronic medical record was accessed to collect clinical information, including the final diagnosis.

Symptom severity was assessed with the Movement Disorder Society Unified Parkinson's Disease Rating Scale part I–III (MDS‐UPDRS), and functional stage with the Hoehn and Yahr scale [21]. Depressive symptoms were evaluated using the Beck Depression Inventory II (BDI‐II), and patients were screened for cognitive impairment with the Montreal Cognitive Assessment (MoCA) [22]. All participants continued their regular medication, including levodopa, during clinical assessment.

The reference standard was the final diagnosis recorded by the treating neurologist, incorporating the DAT SPECT result obtained as part of routine care. If no final diagnosis was documented, the treating neurologist was contacted or the case reviewed by two movement disorder specialists (RdB, 20 years of experience, and MB, 16 years of experience) to reach consensus on whether the parkinsonism was neurodegenerative or non‐neurodegenerative.

DAT SPECT scan information was collected from the medical records, including the qualitative interpretation by the nuclear medicine physician and semi‐quantitative uptake ratios comparing the uptake of the radiotracer in the striatum with the occipital cortex. Because scans were acquired on different SPECT systems, uptake ratios could not be compared across patients. In cases with borderline or inconclusive results, two blinded nuclear medicine physicians (EvdG, 8 years of experience and JB, 30 years of experience) reviewed the scans and reached consensus on whether the DAT SPECT was normal or abnormal.

PD typically begins asymmetrically, affecting one side of the brain more than the other, particularly in the early stages [23]. Therefore, results were evaluated in both the most affected hemisphere (MAH), defined as the side with the lowest uptake ratio of the putamen on the DAT SPECT scan, and the contralateral least affected hemisphere (LAH).

### 2.4 Statistical Analysis

For each HC, the coefficient of variation (CoV) was computed region‐wise as a measure of repeatability for the cortical GM, cerebral WM, thalamus, putamen, caudate nucleus, and globus pallidus. We defined CoV < 1% as excellent, 1% ≤ CoV ≤ 5% as good, 5% ≤ CoV ≤ 10% as acceptable, and CoV ≥ 10% as poor. In the absence of standardized international guidelines for these metrics, we have adopted conservative thresholds based on [24].

Independent samples *t*‐tests were performed to assess differences in T_1_‐values and T_2_‐values in each ROI between patients with neurodegenerative versus non‐neurodegenerative parkinsonism. The distributions were visually assessed for normality. The homogeneity of the variances of the distributions was assessed using Levene's test (*α* = 0.05). In case of unequal variances, Welch's *t*‐test was used, which has been shown to remain robust when variances are unequal [25]. *p* values < 0.05 were considered significant. Cohen's D was computed to test for effect size in regions with significant differences: Cohen's D < 0.5, small effect size; Cohen's D 0.5–0.8, medium effect size; Cohen's D > 0.8, large effect size [26]. Bootstrapping with 2000 samples was performed to determine 95% confidence intervals (CI) of Cohen's D.

The same tests were used to compare T_1_ and T_2_ values of patients with PD and patients with other causes of disease (including neurodegenerative causes), and to compare T_1_ and T_2_ values of patients with PD and patients with non‐neurodegenerative disease (thus excluding other neurodegenerative diseases from the first group).

Given the explorative nature of the current study and the focus on characterizing population‐level differences using a multi‐2D MR‐STAT protocol, no corrections for multiple comparisons were applied to the results in this section. For completeness, in Supporting Information: 2, *p* values corrected for false discovery rate using the Benjamini–Hochberg procedure are provided. These corrections were performed separately for each hemisphere and parameter (11 ROIs per set), reflecting the different biological mechanisms underlying T_1_ and T_2_ and the a priori hypothesis that effects would occur in the MAH.

## 3 Results

### 3.1 Patient Population

One hundred and nineteen patients were enrolled in this study between July 2022 and May 2024. After excluding patients with an uncertain diagnosis (*n* = 1), scans with artifacts (*n* = 5), misalignment in MPRAGE/MR‐STAT registration (*n* = 3) and missing ROIs (*n* = 1), 109 participants remained for final analysis (mean age 66.8 years; 75 males). Of these participants, 52 received a final diagnosis of dopaminergic neurodegenerative parkinsonism, including 48 with PD. The remaining 57 participants were diagnosed with a non‐neurodegenerative cause of parkinsonism. An overview of diagnostic classifications is provided in Table 2. Participants with non‐neurodegenerative parkinsonism had significantly higher scores on the MDS‐UPDRS I, and lower scores on the MoCA, compared to those with neurodegenerative parkinsonism (Table 3). MDS‐UPDRS II‐III scores were also higher in the non‐neurodegenerative group, although this difference did not reach significance. Other characteristics did not differ significantly between groups.

**TABLE 2:** Overview of diagnosis groups for patients with neurodegenerative and non‐neurodegenerative parkinsonism.

Neurodegenerative parkinsonism (*n* = 52)		Non‐neurodegenerative parkinsonism (*n* = 57)	
Parkinson's disease	48	Tremor	17
Multiple system atrophy	1	Functional neurological disorder	8
Corticobasal syndrome	2	Drug‐induced parkinsonism	7
Dementia with Lewy bodies	1	Vascular parkinsonism	3
		Unclassified non‐neurodegenerative parkinsonism	22

*Note:* There were 22 “unclassified non‐neurodegenerative parkinsonism” cases in whom a neurodegenerative cause was initially considered. However, after review of the full clinical course (including clinical follow‐up, neurological assessment, and levodopa response) and DAT SPECT findings, a neurodegenerative cause was dismissed.

**TABLE 3:** Patient characteristics.

	Neurodegenerative parkinsonism (mean)	Non‐neurodegenerative parkinsonism (mean)	*p*
Age (years)	65.4	68.1	0.189
Sex M/F (percentage male)	39/12 (75%)	36/21 (63%)	0.183
Time between DAT SPECT and MRI scan (days)	635.96	674.61	0.511
MDS‐UPDRS I	10.5	12.9	0.048
MDS‐UPDRS II	8.2	10.9	0.053
MDS‐UPDRS III	22.3	26.6	0.066
H&Y 0 (number of patients)	1	2	
H&Y 1 (number of patients)	5	1	
H&Y 2 (number of patients)	36	31	
H&Y 3 (number of patients)	6	19	
H&Y4 (number of patients)	0	1	
BDI‐II	9.6	10.5	0.725
MoCA	25.4	23.6	0.010

Abbreviations: BDI = beck depression inventory II; H&Y = Hoehn and Yahr; MDS UPDRS = movement disorder society unified Parkinson's disease rating scale; MoCA = montreal cognitive assessment (MoCA) to screen for cognitive problems.

### 3.2 In Vivo Repeatability

**FIGURE 2:**
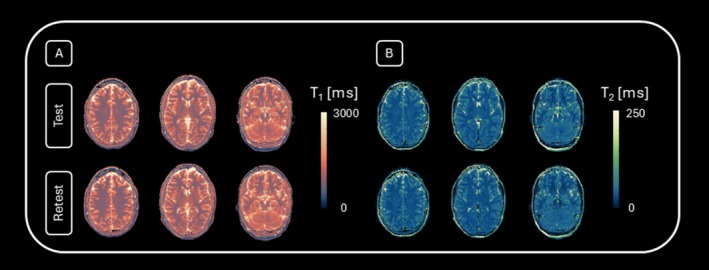
Example quantitative parameter maps of a healthy control who was scanned twice for the repeatability analysis. (A) T_1_ maps. (B) T_2_ maps.

Figure 2 shows quantitative parameter maps at three representative levels of the brain of an HC who was scanned twice using the multi‐2D MR‐STAT protocol. In the cohort of HCs, mean (±sd) T_1_ was 1447 (±34) ms in GM, 1016 (±16) ms in WM, 1206 (±36) ms in the thalamus, 1224 (±29) ms in the putamen, 1293 (±34) ms in the caudate nucleus, and 1088 (±41) ms in the globus pallidus. Mean (±sd) T_2_ was 59 (±3) ms in GM and 42 (±1) ms in WM, 45 (±2) ms in the thalamus, 44 (±2) ms in the putamen, 50 (±2) ms in the caudate nucleus, and 32 (±1) in the globus pallidus. Tables 4 and 5 show the CoVs of T_1_‐ and T_2_‐values, respectively. For T_1_, the mean CoV ranged from 0.5% (WM) to 1.7% (thalamus). For T_2_, the mean CoV ranged from 1.5% (WM) to 2.7% (GM). Repeatability was higher for T_1_ than for T_2_, and there were higher measures of repeatability in WM than in GM for both T_1_ and T_2_. Overall, the repeatability was good or excellent.

**TABLE 4:** Coefficient of variation (CoV) of T_1_ values in healthy controls.

Volunteer	CoV T_1_ GM	CoV T_1_ WM	CoV T_1_ thalamus	CoV T_1_ putamen	CoV T_1_ caudate nucleus	CoV T_1_ globus pallidus
1	3.6%	1.9%	0.3%	1.4%	1.8%	0.3%
2	1.3%	0.9%	5.3%	0.3%	1.3%	0.8%
3	< 0.1%	0.3%	0.8%	0.5%	0.3%	1.6%
4	2.3%	1.3%	3.9%	1.4%	1.6%	1.8%
5	0.7%	0.2%	3.6%	3.3%	3.1%	5.5%
6	0.3%	0.1%	< 0.1%	< 0.1%	0.3%	0.6%
7	0.2%	0.2%	0.2%	0.1%	0.6%	0.3%
8	0.1%	< 0.1%	< 0.1%	0.1%	< 0.1%	< 0.1%
9	< 0.1%	< 0.1%	1.4%	0.9%	1.4%	2.0%
10	0.3%	< 0.1%	1.2%	0.2%	1.1%	0.4%
Mean	0.9%	0.5%	1.7%	0.8%	1.2%	1.3%

Abbreviations: CoV = coefficient of variation; GM = gray matter; WM = white matter.

**TABLE 5:** Coefficient of variation (CoV) of T_2_ values in healthy controls.

Volunteer	CoV T_2_ GM	CoV T_2_ WM	CoV T_2_ thalamus	CoV T_2_ putamen	CoV T_2_ caudate nucleus	CoV T_2_ globus pallidus
1	5.4%	0.9%	1.6%	3.4%	1.5%	2.3%
2	2.0%	0.5%	1.6%	1.7%	1.4%	< 0.1%
3	0.7%	1.3%	4.6%	3.1%	5.4%	6.7%
4	8.4%	1.3%	< 0.1%	4.8%	1.4%	4.6%
5	2.8%	2.3%	4.8%	4.8%	4.2%	4.3%
6	0.1%	< 0.1%	< 0.1%	< 0.1%	1.5%	< 0.1%
7	0.9%	0.4%	< 0.1%	< 0.1%	< 0.1%	2.3%
8	0.3%	0.2%	1.5%	< 0.1%	< 0.1%	< 0.1%
9	2.4%	3.8%	6.3%	2.9%	4.2%	2.2%
10	3.7%	4.2%	3.1%	3.1%	2.8%	2.2%
Mean	2.7%	1.5%	2.4%	2.4%	2.3%	2.5%

Abbreviations: CoV = coefficient of variation; GM = gray matter; WM = white matter.

### 3.3 Imaging Biomarkers of Neurodegenerative Versus Non‐Neurodegenerative Patients

**FIGURE 3:**
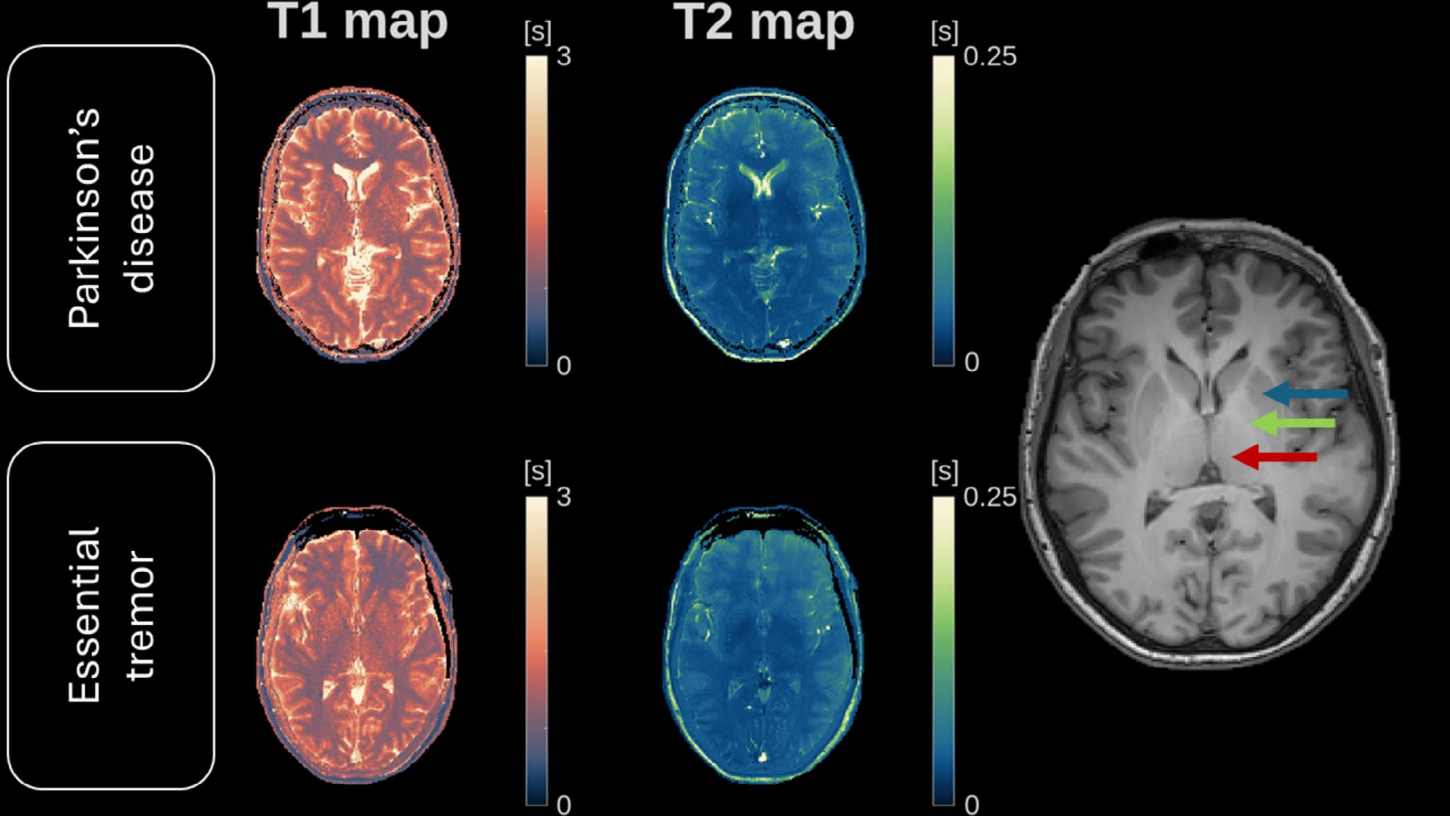
On the left: Example quantitative parameter maps of a patient with neurodegenerative parkinsonism (PD, top), and example quantitative parameter maps of a patient with non‐neurodegenerative parkinsonism (essential tremor, bottom). On the right: A schematic overview of the locations where significant differences were found: The thalamus (red arrow), the putamen (blue arrow) and the globus pallidus (green arrow).

Figure 3 shows example quantitative parameter maps of a patient with neurodegenerative parkinsonism (i.e., PD) and a patient with non‐neurodegenerative parkinsonism (i.e., essential tremor). Table 6 summarizes the regions of the MAH where significant differences were observed between neurodegenerative and non‐neurodegenerative patients. Table 7 summarizes the regions of the less affected hemisphere (LAH) where significant differences were observed. Overall, only differences in T_1_‐values were significant. Differences in T_2_‐values were not statistically significant. There was significant T_1_‐shortening in the thalamus, globus pallidus, both the internal and external globus pallidus and in the anteromedial and centromedial putamen segment. All other regions did not show significant differences. These results can be found in Supporting Information: 4. Notably, higher degrees of T_1_‐shortening, and the strongest effect sizes were found in the MAH. The Cohen's D of the thalamus (0.635, 95% CI = [0.251, 1.016]), globus pallidus (0.508, 95% CI = [0.129, 0.887]) and internal globus pallidus (0.603, 95% CI = [0.220, 0.983]) reflect a medium effect size. For all other regions with significant differences, the effect sizes were small (< 0.5). Comparing PD versus all other causes of parkinsonism, and comparing PD versus non‐neurodegenerative causes of parkinsonism generally showed similar trends in T_1_ shortening, and did not reveal other regions with significant differences (Supporting Information: 3).

**TABLE 6:** Significant T_1_ differences in the most affected hemisphere, comparing neurodegenerative and non‐neurodegenerative CUPS patients.

Parameter	Region	*p*	Mean (SD) [ms]	Cohen's D [95% CI]
T_1_	Thalamus	**0.0012**	Deg: 1268 (60) Nnd: 1310 (70)	**0.635 [0.251, 1.016]**
T_1_	Globus pallidus	**0.0087**	Deg: 1167 (75) Nnd: 1206 (77)	**0.508 [0.129, 0.887]**
T_1_	Centromedial putamen partition	0.0206	Deg: 1273 (60) Nnd: 1306 (85)	0.447 [0.069, 0.824]
T_1_	Internal globus pallidus	**0.0020**	Deg: 1150 (86) Nnd: 1205 (94)	**0.603 [0.220, 0.983]**
T_1_	External globus pallidus	0.0330	Deg: 1183 (70) Nnd: 1215 (83)	0.411 [0.033, 0.787]

*Note: p* values in bold indicate significance after multiple testing correction. Cohen's D in bold indicates at least a medium effect size.

Abbreviations: Deg = neurodegenerative; Nnd = non‐neurodegenerative.

**TABLE 7:** Significant T_1_ differences in the less affected hemisphere, comparing neurodegenerative and non‐neurodegenerative CUPS patients.

Parameter	Region	*p*	Mean (SD) [ms]	Cohen's D [95% CI]
T_1_	Thalamus	0.0315	Deg: 1268 (57) Nnd: 1299 (74)	0.476 [0.097, 0.853]
T_1_	Globus pallidus	0.0138	Deg: 1174 (69) Nnd: 1207 (86)	0.415 [0.037, 0.791]
T_1_	Anteromedial putamen partition	0.0437	Deg: 1285 (48) Nnd: 1311 (82)	0.388 [0.011, 0.764]
T_1_	External globus pallidus	0.0309	Deg: 1183 (70) Nnd: 1215 (83)	0.416 [0.038, 0.792]

*Note: p* values in bold indicate significance after multiple testing correction. Cohen's D in bold indicates at least a medium effect size.

Abbreviations: Deg = neurodegenerative; Nnd = non‐neurodegenerative.

## 4 Discussion

This study investigated relaxometry differences between CUPS patients with dopaminergic neurodegenerative versus non‐neurodegenerative parkinsonism. In both the MAH and LAH, significantly lower T_1_‐values were found in (subdivisions of) the thalamus, globus pallidus, and putamen. Effect sizes were generally larger in the MAH compared to the LAH. Quantitative T_1_‐values have the potential to aid in differentiating neurodegenerative from non‐neurodegenerative parkinsonism.

### 4.1 In Vivo Repeatability

T_1_‐ and T_2_‐values of brain regions in HCs were in similar ranges to those previously reported [27, 28, 29]. A high degree of repeatability was found for the multi‐2D MR‐STAT protocol, similar to that observed for other fast 2D multiparametric relaxometry protocols [24, 30]. The high repeatability provided a robust foundation for comparing patients with neurodegenerative versus non‐neurodegenerative parkinsonism.

### 4.2 Imaging Biomarkers of Neurodegenerative Versus Non‐Neurodegenerative Patients

In this study, comparisons were made between patients with parkinsonism with and without dopaminergic neurodegeneration, with both groups having similar demographics and clinical characteristics, including age, sex distribution and symptom severity. Although the non‐neurodegenerative group showed lower MoCA scores and higher MDS‐UPDRS I scores, these differences likely reflect the clinical heterogeneity of CUPS populations, or the continued use of symptomatic treatment, including levodopa, during clinical assessment. We therefore decided to not adjust T_1_ values for these measures. T_1_‐shortening was observed in the thalamus, putamen and globus pallidus in neurodegenerative CUPS patients, compared to those with non‐neurodegenerative CUPS. T_1_‐shortening in these regions has previously been associated with microstructural changes characteristic of neurodegenerative PD, such as increased iron depositions, dopaminergic neuron loss and microglial activation [31]. While these studies have reported T_1_‐shortening in various brain regions in PD patients relative to HC [8, 9, 10, 11, 12], the current study extends this observation by demonstrating similar differences within a CUPS population. Whereas the globus pallidus has been previously identified as a region with T_1_‐shortening in PD [8], the current study also showed significant T_1_ differences in other brain regions. While the previous studies did not directly compare neurodegenerative versus non‐neurodegenerative CUPS patients, our study is consistent with previous studies in demonstrating T_1_‐shortening suggesting that there may be increased iron depositions in patients with neurodegenerative parkinsonism compared to non‐neurodegenerative parkinsonism. No significant differences in mean T_2_‐values were found, potentially due to a lower degree of repeatability of T_2_‐measurements than T_1_‐measurements. The higher degree of noise in the T_2_‐measurements relative to the T_1_‐measurements could also have contributed. Both repeatability and noise make differentiation based purely on quantitative T_2_‐values more challenging. Previous T_2_ studies in parkinsonian disorders have yielded mixed results, with some reporting clear nigral or basal‐ganglia abnormalities on T_2_ maps [32] while others found only modest or no significant T_2_ differences between patients and controls [33, 34]. Possibly, subtle pathological changes in early‐stage neurodegenerative parkinsonism may not produce substantial alterations in tissue water content that would be reflected in T_2_, whereas the same processes, including microglial activation, neuronal loss, and myelin changes might produce more prominent T_1_ alterations [33]. Also, the substantia nigra, where T_2_/T_2_* changes are most consistently reported in PD, were not assessable with the current 2D MR‐STAT protocol due to limited resolution. Therefore, our negative T_2_ findings apply only to larger subcortical regions and cannot be taken as evidence that T_2_‐based values are not significant in other regions like the substantia nigra.

The largest T_1_ differences were found in the MAH. This may be explained by the lateralized nature of neurodegenerative parkinsonism [35]. These findings are consistent with previous studies, which reported lateralized T_1_‐effects [10, 11, 12], though one study did not observe this pattern [8]. A potential explanation for this discrepancy is differences in the mean disease duration among studies. In the current study, there was a mean time of 1.8 years between the clinical DAT SPECT, which was part of the diagnostic workup, and the acquisition of the MRI scan. Hence, it included a cohort of early‐stage patients. In previous studies where a lateralized effect was found, the mean disease duration was approximately 3.5 to 6 years [7, 8, 9], whereas in Keil et al., where the effect was not observed, the mean disease duration was approximately 8 years [5]. Given the progressive nature of neurodegenerative parkinsonism, it may be plausible that with longer disease duration, the LAH becomes increasingly involved, thereby reducing the apparent asymmetry.

### 4.3 Future Perspectives

The current study showed moderate effect sizes when comparing several brain regions of neurodegenerative versus non‐neurodegenerative parkinsonism patients. However, the current MR‐STAT acquisition and reconstruction did not account for magnetization transfer (MT) effects, which diminish in patients with neurodegenerative parkinsonism in various brain regions [36, 37, 38, 39] and can lengthen apparent T_1_‐ and T_2_‐values of brain tissue [40]. Correcting for MT effects during reconstruction or suppressing macromolecular signal contributions during acquisition may thus lengthen T_1_‐ and T_2_‐values in non‐neurodegenerative parkinsonism patients more than in neurodegenerative parkinsonism patients, potentially increasing T_1_‐differences and potentially showing T_2_‐differences between the groups in certain regions. Future studies using relaxometry should therefore consider incorporating MT correction or suppression strategies [41] to improve sensitivity to neurodegenerative changes.

In this study, differences in relaxation times in cerebral structures were explored using multi‐2D MR‐STAT. However, the spatial resolution of this technique was limited. Several studies have suggested investigating small brainstem regions, such as the substantia nigra [10, 11]. However, due to the slice thickness (3 mm) and interslice gap (1.5 mm) of the 2D MR‐STAT sequence used in this study, reliable assessment of such small structures was unfeasible. Recently, a 3D MR‐STAT sequence with 1 mm isotropic resolution was introduced [29], which enables more detailed investigations of these small brain regions and would allow further exploration of differences in T_1_ and T_2_ relaxation times in important regions such as the substantia nigra and locus coeruleus. Furthermore, the 3D protocol has a higher signal‐to‐noise ratio (SNR) than the 2D protocol, possibly leading to better differentiation and thus improved predictive power [42]. Another potential application of the higher‐resolution 3D MR‐STAT [29] is to longitudinal studies, where it may offer an opportunity to monitor disease progression of PD and related disorders in a shorter scan time. Whereas previously, T_1_‐ and B_1_‐mapping have been performed with a scan time of approximately 14 min [43], a scan time of < 6 min (including B_1_ field calibration) using 3D MR‐STAT may result in a more patient‐friendly alternative and would additionally include T_2_‐mapping. As such, 3D MR‐STAT is a promising tool for both clinical research and future longitudinal imaging protocols to better understand disease progression and effects of therapy in neurodegenerative disorders like PD.

Additionally, the neurodegenerative group was predominantly Parkinson's disease, whereas other neurodegenerative etiologies were rare and no patients ultimately received a diagnosis of progressive supranuclear palsy (PSP). Therefore, our findings are principally generalizable to PD‐predominant dopaminergic neurodegenerative parkinsonism rather than to specific atypical syndromes such as PSP. Future studies including larger numbers of atypical parkinsonian syndromes, such as PSP and multiple system atrophy, are needed to further research T_1_ and T_2_ relaxation time alterations across the broader spectrum of parkinsonian disorders.

## 5 Limitations

The retrospective identification of patients who underwent DAT SPECT as part of their routine clinical diagnostic work‐up because of CUPS resulted in a mean delay of nearly 2 years between DAT SPECT and MRI scans. In neurodegenerative diseases like PD, progression over time may have influenced the imaging results. However, a previous study has shown no significant group differences in annualized percent change rates of T_1_ in the caudate nucleus, putamen, and thalamus [43] in PD patients compared to HC. Therefore, the potential impact of this delay on the current results may be negligible. While the retrospective design introduced this limitation, it allowed inclusion of a large cohort of patients with CUPS, enabling an evaluation of the potential clinical utility of relaxometry‐based methods.

The reference standard in this study was the final diagnosis established by the treating neurologist, incorporating DAT SPECT findings. Since pathological confirmation was not feasible, this approach provided the best available alternative. Although a follow‐up period could have improved diagnostic certainty, DAT SPECT shows high accuracy (up to 98%) for differentiating neurodegenerative from non‐neurodegenerative parkinsonism [5].

The repeatability analysis was performed in health controls. However, the age of the patient cohort was higher, potentially leading to a higher degree of motion burden and pulsatility [44, 45], which could potentially influence the degree of repeatability. The findings from the repeatability analysis may therefore not fully generalize to the patient cohort.

Finally, as discussed in the previous section, spatial resolution was limited in this study and no account was taken of MT effects.

## 6 Conclusion

This study assessed the repeatability of MR‐STAT and investigated differences in T_1_‐ and T_2_‐values between cortical brain structures in neurodegenerative versus non‐neurodegenerative CUPS patients. It showed excellent repeatability for T_1_ and T_2_ in HC, and significant T_1_‐shortening in neurodegenerative CUPS patients in (subdivisions of) the thalamus, putamen, and globus pallidus, with the strongest effects in the MAH.

## Funding

The study was investigator initiated and partly funded by Heuron Co. Ltd. Data acquisition and analyses were performed without the involvement of Heuron Co. Ltd. Heuron Co. Ltd. did not have influence on the reported results. This research was partly supported by the Netherlands Organization for Scientific Research (NWO), Grant 17986.

## Conflicts of Interest

J.B. has performed contract research for Heuron Inc., J.B. is a consultant for GE Healthcare, and is an image reader for IXICO. All support was paid to the institution. EvdG has received research support from NWO, ZonMw, Hersenstichting, Alzheimer Nederland, Health~Holland and KWF. EvdG has performed contract research for Heuron Inc., AC Immune and Roche. EvdG has a consultancy agreement with IXICO and Life Molecular Imaging for reading PET scans. All support was paid to the institution.

## Supporting information


**Figure S1:** The optimized RF flip angle train used for experiments 1 and 2.
**Tabel S1:** Benjamini–Hochberg corrected *p* values for the most affected hemisphere comparing patients with neurodegenerative parkinsonism versus patients with non‐neurodegenerative parkinsonism. Bold indicates significance after multiple testing correction.
**Tabel S2:** Benjamini–Hochberg corrected *p* values for the less affected hemisphere. Bold indicates significance after multiple testing correction.
**Tabel S3:** Statistical comparison of the MAH for PD versus other causes of parkinsonism. Bold indicates significance after multiple testing correction.
**Tabel S4:** Statistical comparison of the LAH for PD versus other causes of parkinsonism. Bold indicates significance after multiple testing correction.
**Tabel S5:** Statistical comparison of the MAH for PD versus non‐neurodegenerative. Bold indicates significance after multiple testing correction.
**Tabel S6:** Statistical comparison of the LAH for PD versus non‐neurodegenerative. Bold indicates significance after multiple testing correction.
**Table S7:** Results from non‐significant regions in the most affected hemisphere, *p* values and Cohen's D.
**Table S8:** Results from non‐significant regions in the less affected hemisphere, *p* values and Cohen's D.

## References

[1] R. B. Postuma , D. Berg , M. Stern , et al., “MDS Clinical Diagnostic Criteria for Parkinson's Disease,” Movement Disorders 30 (2015): 1591–1601. 26474316 10.1002/mds.26424

[2] J. H. Kordower , C. W. Olanow , H. B. Dodiya , et al., “Disease Duration and the Integrity of the Nigrostriatal System in Parkinson's Disease,” Brain 136 (2013): 2419–2431. 23884810 10.1093/brain/awt192 PMC3722357

[3] S. H. Fox , D. G. Luca , R. B. Postuma , et al., “Revisiting the 2015 MDS Diagnostic Criteria for Parkinson Disease: Insights From Autopsy‐Confirmed Cases,” npj Parkinson's Disease 11 (2025): 360. 10.1038/s41531-025-01206-6 PMC12748991 41390531

[4] F. J. A. Meijer , B. Goraj , B. R. Bloem , and R. A. J. Esselink , “Clinical Application of Brain MRI in the Diagnostic Work‐Up of Parkinsonism,” Journal of Parkinson's Disease 7 (2017): 211–217. 10.3233/JPD-150733 PMC5438480 28282809

[5] S. R. Suwijn , C. J. van Boheemen , R. J. de Haan , G. Tissingh , J. Booij , and R. M. de Bie , “The Diagnostic Accuracy of Dopamine Transporter SPECT Imaging to Detect Nigrostriatal Cell Loss in Patients With Parkinson's Disease or Clinically Uncertain Parkinsonism: A Systematic Review,” EJNMMI Research 5 (2015): 12. 25853018 10.1186/s13550-015-0087-1 PMC4385258

[6] M. J. Armstrong and M. S. Okun , “Diagnosis and Treatment of Parkinson Disease,” JAMA 323 (2020): 548. 32044947 10.1001/jama.2019.22360

[7] E. Tolosa , A. Garrido , S. W. Scholz , and W. Poewe , “Challenges in the Diagnosis of Parkinson's Disease,” Lancet Neurology 20 (2021): 385–397. 33894193 10.1016/S1474-4422(21)00030-2 PMC8185633

[8] V. C. Keil , S. P. Bakoeva , A. Jurcoane , et al., “A Pilot Study of Magnetic Resonance Fingerprinting in Parkinson's Disease,” NMR in Biomedicine 33 (2020): e4389. 32783321 10.1002/nbm.4389

[9] E. Drori , S. Berman , and A. A. Mezer , “Mapping Microstructural Gradients of the Human Striatum in Normal Aging and Parkinson's Disease,” Science Advances 8 (2022): eabm1971. 35857492 10.1126/sciadv.abm1971 PMC9286505

[10] M. Duan , R. Pan , Q. Gao , et al., “A Rapid Multi‐Parametric Quantitative MR Imaging Method to Assess Parkinson's Disease: A Feasibility Study,” BMC Medical Imaging 24 (2024): 58. 38443786 10.1186/s12880-024-01229-0 PMC10916029

[11] S. Baudrexel , L. Nürnberger , U. Rüb , et al., “Quantitative Mapping of T1 and T2* Discloses Nigral and Brainstem Pathology in Early Parkinson's Disease,” NeuroImage 51 (2010): 512–520. 20211271 10.1016/j.neuroimage.2010.03.005

[12] M. Klietz , M. H. Elaman , N. Mahmoudi , et al., “Cerebral Microstructural Alterations in Patients With Early Parkinson's Disease Detected With Quantitative Magnetic Resonance Measurements,” Frontiers in Aging Neuroscience 13 (2021): 763331. 34790113 10.3389/fnagi.2021.763331 PMC8591214

[13] A. Sbrizzi , O. van der Heide , M. Cloos , et al., “Fast Quantitative MRI as a Nonlinear Tomography Problem,” Magnetic Resonance Imaging 46 (2018): 56–63. 29103975 10.1016/j.mri.2017.10.015 PMC6080622

[14] J. P. D. Kleinloog , S. Mandija , F. D'Agata , et al., “Synthetic MRI With Magnetic Resonance Spin TomogrAphy in Time‐Domain (MR‐STAT): Results From a Prospective Cross‐Sectional Clinical Trial,” Journal of Magnetic Resonance Imaging 57 (2023): 1451–1461. 36098348 10.1002/jmri.28425

[15] M. B. Schilder , S. Mandija , S. M. Jacobs , et al., “Fast Whole Brain Relaxometry With Magnetic Resonance Spin TomogrAphy in Time‐Domain (MR‐STAT) at 3 T: A Retrospective Cohort Study,” Magnetic Resonance Materials in Physics, Biology and Medicine 38 (2025): 333–345. 10.1007/s10334-025-01237-3 PMC11914305 40035911

[16] M. Fuderer , O. van der Heide , H. Liu , C. A. T. van den Berg , and A. Sbrizzi , “Efficient Performance Analysis and Optimization of Transient‐State Sequences for Multiparametric Magnetic Resonance Imaging,” NMR in Biomedicine 36 (2023): e4864. 36321222 10.1002/nbm.4864 PMC10078474

[17] O. van der Heide , C. A. T. van den Berg , and A. Sbrizzi , “GPU‐Accelerated Bloch Simulations and MR‐STAT Reconstructions Using the Julia Programming Language,” Magnetic Resonance in Medicine 92 (2024): 618–630. 38441315 10.1002/mrm.30074

[18] K. Nehrke and P. Börnert , “DREAM—A Novel Approach for Robust, Ultrafast, Multislice *B* _1_ Mapping,” Magnetic Resonance in Medicine 68 (2012): 1517–1526. 22252850 10.1002/mrm.24158

[19] P. Coupé , B. Mansencal , M. Clément , et al., “AssemblyNet: A Large Ensemble of CNNs for 3D Whole Brain MRI Segmentation,” NeuroImage 219 (2020): 117026. 32522665 10.1016/j.neuroimage.2020.117026

[20] A. D. Dorval , A. Muralidharan , A. L. Jensen , K. B. Baker , and J. L. Vitek , “Information in Pallidal Neurons Increases With Parkinsonian Severity,” Parkinsonism & Related Disorders 21 (2015): 1355–1361. 26433544 10.1016/j.parkreldis.2015.09.045 PMC4631644

[21] C. G. Goetz , S. Fahn , P. Martinez‐Martin , et al., “Movement Disorder Society‐Sponsored Revision of the Unified Parkinson's Disease Rating Scale (MDS‐UPDRS): Process, Format, and Clinimetric Testing Plan,” Movement Disorders 22 (2007): 41–47. 17115387 10.1002/mds.21198

[22] Z. S. Nasreddine , N. A. Phillips , V. Bédirian , et al., “The Montreal Cognitive Assessment, MoCA: A Brief Screening Tool for Mild Cognitive Impairment,” Journal of the American Geriatrics Society 53 (2005): 695–699. 15817019 10.1111/j.1532-5415.2005.53221.x

[23] P. A. Kempster , W. R. Gibb , G. M. Stern , and A. J. Lees , “Asymmetry of Substantia Nigra Neuronal Loss in Parkinson's Disease and Its Relevance to the Mechanism of Levodopa Related Motor Fluctuations,” Journal of Neurology, Neurosurgery, and Psychiatry 52 (1989): 72–76. 2709038 10.1136/jnnp.52.1.72 PMC1032660

[24] G. Buonincontri , L. Biagi , A. Retico , et al., “Multi‐Site Repeatability and Reproducibility of MR Fingerprinting of the Healthy Brain at 1.5 and 3.0 T,” NeuroImage 195 (2019): 362–372. 30923028 10.1016/j.neuroimage.2019.03.047

[25] H. O. Posten , “Robustness of the Two‐Sample T‐Test,” in Robustness of Statistical Methods and Nonparametric Statistics (Springer Netherlands, 1984), 92–99.

[26] J. Cohen , “A Power Primer,” Psychological Bulletin 112 (1992): 155–159. 19565683 10.1037//0033-2909.112.1.155

[27] J. Z. Bojorquez , S. Bricq , C. Acquitter , F. Brunotte , P. M. Walker , and A. Lalande , “What Are Normal Relaxation Times of Tissues at 3T?,” Magnetic Resonance Imaging 35 (2017): 69–80. 27594531 10.1016/j.mri.2016.08.021

[28] J. Y. Choi , S. Hu , T.‐Y. Su , et al., “Normative Quantitative Relaxation Atlases for Characterization of Cortical Regions Using Magnetic Resonance Fingerprinting,” Cerebral Cortex 33 (2023): 3562–3574. 35945683 10.1093/cercor/bhac292 PMC10068276

[29] H. Liu , E. Versteeg , M. Fuderer , et al., “Time‐Efficient, High‐Resolution 3T Whole‐Brain Relaxometry Using Cartesian 3D MR Spin TomogrAphy in Time‐Domain (MR‐STAT) With Cerebrospinal Fluid Suppression,” Magnetic Resonance in Medicine 93 (2025): 2008–2019. 39607873 10.1002/mrm.30384 PMC11893030

[30] L. Nunez‐Gonzalez , G. Kotek , P. A. Gómez , et al., “Accuracy and Repeatability of QRAPMASTER and MRF‐vFA,” Magnetic Resonance Imaging 83 (2021): 196–207. 34506911 10.1016/j.mri.2021.09.004

[31] W. Zhang , Z. Yan , J. Gao , et al., “Role and Mechanism of Microglial Activation in Iron‐Induced Selective and Progressive Dopaminergic Neurodegeneration,” Molecular Neurobiology 49 (2014): 1153–1165. 24277523 10.1007/s12035-013-8586-4 PMC4878835

[32] A. Antonini , K. L. Leenders , D. Meier , W. H. Oertel , P. Boesiger , and M. Anliker , “T _2_ Relaxation Time in Patients With Parkinson's Disease,” Neurology 43 (1993): 697–700. 8469325 10.1212/wnl.43.4.697

[33] J. Vymazal , A. Righini , R. A. Brooks , et al., “T1 and T2 in the Brain of Healthy Subjects, Patients With Parkinson Disease, and Patients With Multiple System Atrophy: Relation to Iron Content,” Radiology 211 (1999): 489–495. 10228533 10.1148/radiology.211.2.r99ma53489

[34] Q. Cao , J. Huang , D. Tang , et al., “Application Value of Multiparametric MRI for Evaluating Iron Deposition in the Substantia Nigra in Parkinson's Disease,” Frontiers in Neurology 13 (2023): 1096966. 36686531 10.3389/fneur.2022.1096966 PMC9846143

[35] R. Djaldetti , I. Ziv , and E. Melamed , “The Mystery of Motor Asymmetry in Parkinson's Disease,” Lancet Neurology 5 (2006): 796–802. 16914408 10.1016/S1474-4422(06)70549-X

[36] N. Tambasco , V. Belcastro , P. Sarchielli , et al., “A Magnetization Transfer Study of Mild and Advanced Parkinson's Disease,” European Journal of Neurology 18 (2011): 471–477. 20722713 10.1111/j.1468-1331.2010.03184.x

[37] Y. Anik , P. Iseri , A. Demirci , S. Komsuoglu , and N. Inan , “Magnetization Transfer Ratio in Early Period of Parkinson Disease,” Academic Radiology 14 (2007): 189–192. 17236991 10.1016/j.acra.2006.11.005

[38] T. Eckert , M. Sailer , J. Kaufmann , et al., “Differentiation of Idiopathic Parkinson's Disease, Multiple System Atrophy, Progressive Supranuclear Palsy, and Healthy Controls Using Magnetization Transfer Imaging,” NeuroImage 21 (2004): 229–235. 14741660 10.1016/j.neuroimage.2003.08.028

[39] H. Hanyu , T. Asano , M. Takasaki , H. Shindo , K. Abe , and H. Sakurai , “Magnetisation Transfer Measurements of the Subcortical Grey and White Matter in Parkinson's Disease With and Without Dementia and in Progressive Supranuclear Palsy,” Neuroradiology 43 (2001): 542–546. 11512582 10.1007/s002340100558

[40] T. Hilbert , D. Xia , K. T. Block , et al., “Magnetization Transfer in Magnetic Resonance Fingerprinting,” Magnetic Resonance in Medicine 84 (2020): 128–141. 31762101 10.1002/mrm.28096 PMC7083689

[41] J. Assländer and S. Flassbeck , “Magnetization Transfer Explains Most of the *T* _1_ Variability in the MRI Literature,” Magnetic Resonance in Medicine 94 (2025): 293–301. 40096551 10.1002/mrm.30451 PMC12021565

[42] H. Liu , O. van der Heide , E. Versteeg , et al., “A Three‐Dimensional Magnetic Resonance Spin Tomography in Time‐Domain Protocol for High‐Resolution Multiparametric Quantitative Magnetic Resonance Imaging,” NMR in Biomedicine 37 (2024): e5050. 37857335 10.1002/nbm.5050

[43] L. Nürnberger , R.‐M. Gracien , P. Hok , et al., “Longitudinal Changes of Cortical Microstructure in Parkinson's Disease Assessed With T1 Relaxometry,” Neuroimage: Clinical 13 (2017): 405–414. 28116233 10.1016/j.nicl.2016.12.025 PMC5226811

[44] C. H. Krag , F. C. Müller , K. L. Gandrup , et al., “Motion Artifacts and Image Quality in Stroke MRI: Associated Factors and Impact on AI and Human Diagnostic Accuracy,” European Radiology 36 (2025): 265–277. 40664863 10.1007/s00330-025-11807-7 PMC12712086

[45] V. Perosa , T. Arts , A. Assmann , et al., “Pulsatility Index in the Basal Ganglia Arteries Increases With Age in Elderly With and Without Cerebral Small Vessel Disease,” American Journal of Neuroradiology 43 (2022): 540–546. 35332021 10.3174/ajnr.A7450 PMC8993201

