# Supplementary material for: Mapping Neurodegenerative Changes in Clinically Uncertain Parkinsonian Syndrome Patients Using Fast MR Spin TomogrAphy in Time‐Domain (MR‐STAT) Relaxometry at 3T
Source: J Magn Reson Imaging. 2026 Apr 25;64(2):429–39. doi: 10.1002/jmri.70349 (PMC13356393; doi:10.1002/jmri.70349)
Supplement: Supplementary file 1 — Figure S1: The optimized RF flip angle train used for experiments 1 and 2. Tabel S1: Benjamini–Hochberg corrected p values for the most affected hemisphere comparing patients with neurodegenerative parkinsonism versus patients with non‐neurodegenerative parkinsonism. Bold indicates significance after multiple testing correction. Tabel S2: Benjamini–Hochberg corrected p values for the less affected hemisphere. Bold indicates significance after multiple testing correction. Tabel S3: Statistical comparison of the MAH for PD versus other causes of parkinsonism. Bold indicates significance after multiple testing correction. Tabel S4: Statistical comparison of the LAH for PD versus other causes of parkinsonism. Bold indicates significance after multiple testing correction. Tabel S5: Statistical comparison of the MAH for PD versus non‐neurodegenerative. Bold indicates significance after multiple testing correction. Tabel S6: Statistical comparison of the LAH for PD versus non‐neurodegenerative. Bold indicates significance after multiple testing correction. Table S7: Results from non‐significant regions in the most affected hemisphere, p values and Cohen's D. Table S8: Results from non‐significant regions in the less affected hemisphere, p values and Cohen's D. [file JMRI-64-429-s001.docx]

# Supplementary Materials 1


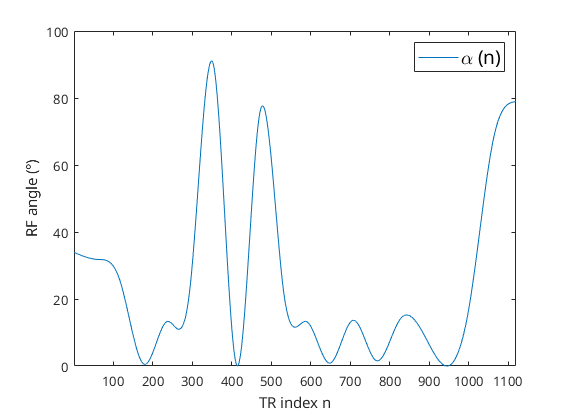


Figure 1: The optimized RF flip angle train used for experiments 1 and 2.

# Supplementary Materials 2

Supplementary Tabel 1: Benjamini-Hochberg corrected p-values for the most affected hemisphere comparing patients with neurodegenerative parkinsonism versus patients with non-neurodegenerative parkinsonism. Bold indicates significance after multiple testing correction.

| Parameter | Region | P-value | Adjusted p-value |
| --- | --- | --- | --- |
| T_1_ | Thalamus | 0.0012 | **0.0110** |
| T_1_ | Globus pallidus | 0.0087 | **0.0319** |
| T_1_ | Centromedial putamen partition | 0.0206 | 0.05665 |
| T_1_ | Internal globus pallidus | 0.0020 | **0.0110** |
| T_1_ | External globus pallidus | 0.0330 | 0.0726 |

Supplementary Tabel 2: Benjamini-Hochberg corrected p-values for the less affected hemisphere. Bold indicates significance after multiple testing correction.

| Parameter | Region | P-value | Adjusted p-value |
| --- | --- | --- | --- |
| T_1_ | Thalamus | 0.0315 | 0.1155 |
| T_1_ | Globus pallidus | 0.0138 | 0.1155 |
| T_1_ | Anteromedial putamen partition | 0.0437 | 0.1202 |
| T_1_ | External globus pallidus | 0.0309 | 0.1155 |

# Supplementary Materials 3

Supplementary Tabel 3: Statistical comparison of the MAH for PD versus other causes of parkinsonism. Bold indicates significance after multiple testing correction.

| Parameter | Region | P-value | Adjusted p-value | Mean (SD) [ms] | Cohen’s D [95% CI] |
| --- | --- | --- | --- | --- | --- |
| T_1_ | Thalamus | 0.0032 | **0.0352** | PD: 1269 (60)  Other: 1307 (71) | 0.577 [0.193, 0.959] |
| T_1_ | Globus pallidus | 0.0250 | 0.0688 | PD: 1169 (75)  Other: 1202 (79) | 0.435 [0.055, 0.811] |
| T_1_ | Centromedial putamen partition | 0.0150 | 0.0550 | PD: 1271 (59)  Other: 1306 (84) | 0.473 [0.091, 0.852] |
| T_1_ | Internal globus pallidus | 0.0096 | 0.0528 | PD: 1153 (84)  Other: 1199 (97) | 0.504 [0.122, 0.884] |

Supplementary Tabel 4: Statistical comparison of the LAH for PD versus other causes of parkinsonism. Bold indicates significance after multiple testing correction.

| Parameter | Region | P-value | Adjusted p-value | Mean (SD) [ms] | Cohen’s D [95% CI] |
| --- | --- | --- | --- | --- | --- |
| T_1_ | Thalamus | 0.0175 | 0.1925 | PD: 1267 (54)  Other: 1298 (75) | 0.461 [0.081, 0.840] |

Supplementary Tabel 5: Statistical comparison of the MAH for PD versus non-neurodegenerative. Bold indicates significance after multiple testing correction.

| Parameter | Region | P-value | Adjusted p-value | Mean (SD) [ms] | Cohen’s D [95% CI] |
| --- | --- | --- | --- | --- | --- |
| T_1_ | Thalamus | 0.0016 | **0.0176** | PD: 1269 (60)  Nnd: 1310 (70) | 0.630 [0.239, 1.019] |
| T_1_ | Globus pallidus | 0.0134 | **0.0434** | PD: 1169 (75)  Nnd: 1206 (77) | 0.489 [0.101, 0.874] |
| T_1_ | Centromedial putamen partition | 0.0158 | **0.0434** | PD: 1271 (59)  Nnd: 1306 (85) | 0.477 [0.089, 0.862] |
| T_1_ | External globus pallidus | 0.0413 | 0.0909 | PD: 1178 (82)  Nnd: 1211 (80) | 0.401 [0.016, 0.785] |
| T_1_ | Internal globus pallidus | 0.0035 | **0.0193** | PD: 1153 (84)  Nnd: 1205 (94) | 0.589 [0.189, 0.967] |

Supplementary Tabel 6: Statistical comparison of the LAH for PD versus non-neurodegenerative. Bold indicates significance after multiple testing correction.

| Parameter | Region | P-value | Adjusted p-value | Mean (SD) [ms] | Cohen’s D [95% CI] |
| --- | --- | --- | --- | --- | --- |
| T_1_ | Thalamus | 0.0129 | 0.1419 | PD: 1267 (54)  Nnd: 1299 (74) | 0.491 [0.104, 0.877] |
| T_1_ | Globus pallidus | 0.0497 | 0.1822 | PD: 1177 (68)  Nnd: 1207 (86) | 0.386 [0.000, 0.769] |
| T_1_ | External globus pallidus | 0.0481 | 0.1822 | Deg: 1183 (70)  Nnd: 1215 (83) | 0.388 [0.003, 0.772] |

# Supplementary Materials 4

*Supplementary Table 7: Results from non-significant regions in the most affected hemisphere, p-values and Cohen’s D*

| Parameter | Region | P-value | Cohen’s D [95% CI] |
| --- | --- | --- | --- |
| T1 | Cortical GM | 0.9605 | -0.010 [-0.382; 0.364] |
| T1 | Cerebral WM | 0.5170 | -0.124 [-0.497; 0.250] |
| T1 | Putamen | 0.0765 | 0.338 [-0.038; 0.713] |
| T1 | Caudate nucleus | 0.1433 | 0.280 [-0.096; 0.654] |
| T1 | Anterior putamen partition | 0.3386 | 0.1181 [-0.193; 0.554] |
| T1 | Anteromedial putamen partition | 0.1317 | 0.287 [-0.088; 0.662] |
| T1 | Posteromedial putamen partition | 0.0832 | 0.330 [-0.046; 0.703] |
| T1 | Posterior putamen partition | 0.7804 | 0.054 [-0.319; 0.427] |
| T2 | Cortical GM | 0.0769 | -0.342 [-0.717 ; 0.035] |
| T2 | Cerebral WM | 0.4739 | -0.137 [-0.510; 0.237] |
| T2 | Thalamus | 0.2184 | 0.237 [-0.138; 0.611] |
| T2 | Putamen | 0.7825 | 0.053 [-0.320; 0.426] |
| T2 | Caudate nucleus | 0.3373 | -0.183 [-0.557; 0.191] |
| T2 | Globus pallidus | 0.1155 | 0.302 [-0.074; 0.676] |
| T2 | Anterior putamen partition | 0.9106 | -0.021 [-0.394; 0.352] |
| T2 | Anteromedial putamen partition | 0.6141 | 0.097 [-0.277; 0.469] |
| T2 | Centromedial putamen partition | 0.5681 | -0.109 [-0.264; 0.482] |
| T2 | Posteromedial putamen partition | 0.7322 | 0.066 [-0.308; 0.439] |
| T2 | Posterior putamen partition | 0.6756 | -0.080 [-0.453; 0.293] |
| T2 | Internal globus pallidus | 0.1362 | 0.286 [0.089; 0.661] |
| T2 | External globus pallidus | 0.1523 | 0.274 [0.101; 0.648] |

*Supplementary Table 8: Results from non-significant regions in the less affected hemisphere, p-values and Cohen’s D*

| Parameter | Region | P-value | Cohen’s D [95% CI] |
| --- | --- | --- | --- |
| T1 | Cortical GM | 0.7788 | -0.054 [-0.427; 0.320] |
| T1 | Cerebral WM | 0.1552 | 0.272 [-0.103; 0.646] |
| T1 | Putamen | 0.0830 | 0.330 [-0.038; 0.713] |
| T1 | Caudate nucleus | 0.5692 | 0.108 [-0.265; 0.481] |
| T1 | Anterior putamen partition | 0.3624 | 0.172 [-0.202; 0.546] |
| T1 | Centromedial putamen partition | 0.2515 | 0.217 [-0.157; 0.591] |
| T1 | Posteromedial putamen partition | 0.0642 | 0.353 [-0.024; 0.728] |
| T1 | Posterior putamen partition | 0.3974 | 0.161 [-0.213; 0.534] |
| T1 | Internal globus pallidus | 0.2502 | 0.222 [-0.153 ;0.596] |
| T2 | Cortical GM | 0.1980 | -0.248 [-0.622; 0.127] |
| T2 | Cerebral WM | 0.8163 | -0.124 [-0.497; 0.250] |
| T2 | Thalamus | 0.2908 | 0.202 [-0.138; 0.611] |
| T2 | Putamen | 0.6091 | 0.098 [-0.276; 0.471] |
| T2 | Caudate nucleus | 0.4510 | -0.145 [-0.518; 0.229] |
| T2 | Globus pallidus | 0.0930 | 0.321 [-0.055; 0.696] |
| T2 | Anterior putamen partition | 0.8546 | 0.035 [-0.338; 0.408] |
| T2 | Anteromedial putamen partition | 0.4989 | 0.130 [-0.244; 0.503] |
| T2 | Centromedial putamen partition | 0.6531 | 0.086 [-0.288; 0.459] |
| T2 | Posteromedial putamen partition | 0.8230 | 0.043 [-0.331; 0.416] |
| T2 | Posterior putamen partition | 0.8338 | 0.040 [-0.333; 0.413] |
| T2 | Internal globus pallidus | 0.1186 | 0.298 [-0.077; 0.672] |
| T2 | External globus pallidus | 0.0936 | 0.321 [-0.055; 0.696] |
